# The genome sequence of the Marbled Maple moth, *Altenia scriptella* (Hübner, 1796) (Lepidoptera: Gelechiidae)

**DOI:** 10.12688/wellcomeopenres.26552.1

**Published:** 2026-07-11

**Authors:** David C. Lees, Natural History Museum Genome Acquisition Lab, Darwin Tree of Life Barcoding Collective, Wellcome Sanger Institute Tree of Life Management, Samples and Laboratory team, Wellcome Sanger Institute Scientific Operations: Sequencing Operations, Wellcome Sanger Institute Tree of Life Core Informatics team, Tree of Life Core Informatics collective, Darwin Tree of Life Consortium

**Affiliations:** 1 Natural History Museum, London, England, UK

**Keywords:** Altenia scriptella, Marbled Maple moth, Horse-shoe Groundling, genome sequence, chromosomal, Lepidoptera

## Abstract

We present a genome assembly from an individual female *Altenia scriptella* (Marbled Maple moth; Arthropoda; Insecta; Lepidoptera; Gelechiidae). The assembly contains two haplotypes with total lengths of 506.56 megabases and 440.34 megabases. Most of haplotype 1 (99.51%) is scaffolded into 33 chromosomal pseudomolecules, including the W and Z sex chromosomes. The assembly also includes two B chromosomes. Haplotype 2 was assembled to scaffold level. The mitochondrial genome has also been assembled, with a length of 15.3 kilobases. This assembly was generated as part of the Darwin Tree of Life project, which produces genomes for eukaryotic species found in Britain and Ireland.

## Species taxonomy

Eukaryota; Opisthokonta; Metazoa; Eumetazoa; Bilateria; Protostomia; Ecdysozoa; Panarthropoda; Arthropoda; Mandibulata; Pancrustacea; Altocrustacea; Allotriocarida; Hexapoda; Insecta; Dicondylia; Pterygota; Neoptera; Eumetabola; Endopterygota; Aparaglossata; Panorpida; Amphiesmenoptera; Lepidoptera; Glossata; Neolepidoptera; Heteroneura; Ditrysia; Gelechioidea; Gelechiidae; Gelechiinae; *Altenia*; *Altenia scriptella* (Hübner, 1796) (NCBI:txid1448893).

## Background

**Figure 1.**
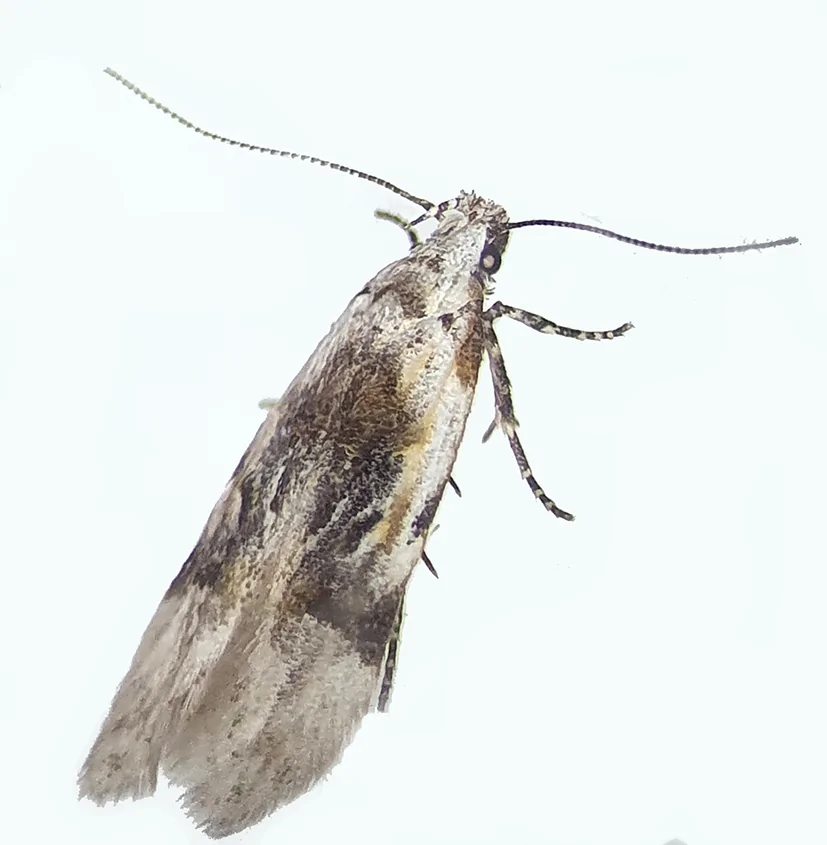
Photograph of the *Altenia scriptella* (ilAltScri1) specimen used for genome sequencing.

*Altenia scriptella* (Hübner, 1796), the Marbled Maple moth, is an elegantly patterned moth in the family Gelechiidae. The male has a forewing length of 6–7 mm ( Sterling *et al.*, 2023) and a wingspan of about 12–15 mm ( Huemer & Karsholt, 2023: 100). The wing pattern is highly variable, but the forewing background colour is generally whitish with fine grey, orange-brown and black speckling. When at rest ( Figure 1), the adult moth exhibits an intricately patterned brownish to blackish blotch from one third to two-thirds along the forewings, outwardly demarcated by a perpendicular black margin terminating with a black mark at two-thirds along the costa; there is a preceding black mark on the costa. This blotch contains up to five patches of readily dehiscent raised scales on each forewing and encompasses a pair of black dashes outlined with pale scales, the innermost of which is straight and slightly oblique to the tergum, the outermost of which is curved towards the costa. The raised scales and a yellowish streak close to the costa distinguish the species from *Carpatolechia alburnella* (Zeller, 1839), sometimes confusable in the UK. In Europe, *A. wagneriella* (Rebel, 1926) is similarly marked but is larger and darker ( Huemer & Karsholt, 2023). The hindwing is grey with a grey fringe. The thorax and head are whitish to greyish, and the legs and labial palps are ringed black and white.

The Marbled Maple Moth likes to rest on tree trunks and is attracted to light at night ( Sterling *et al.*, 2023). This single-brooded moth flies from mid-May to late July with a peak in mid-June ( Norfolk Moths, 2025). In the UK it is very local, currently with few annual records, occurring in the southern half of England, from Somerset to East Anglia and sparsely in Herefordshire and Worcestershire, but without reliable records further north, being absent from Scotland and Ireland. In the UK it is rated as Nationally Scarce A ( Gelechiidae Recording Scheme, 2026) and considered to be declining ( Bland *et al.*, 2002), while there are 504 records in Europe ( GBIF Secretariat, 2025) from Portugal to Central Asia and from Italy to southern Scandinavia.

The Marbled Maple Moth feeds as a larva within an upward fold of a spun leaf of Field Maple *Acer campestre* L. stitched at the margins with bundles of silk, browsing the upper surface of the parenchyma. In Europe, *A. pseudoplatanus* L. and *A. platanoides* L. are also mentioned as foodplants ( Huemer & Karsholt, 2023: 101). The whitish green caterpillar with black pinacula can be found in August and September, favouring lower branches of saplings at wood margins ( Bland *et al.*, 2002). The red-brown pupa is contained in a flimsy white cocoon amongst detritus ( Bland *et al.*, 2002).

On BOLD, as checked on 28 November 2025, the DNA barcode from the mitochondrial genome assembly (OZ350385.1) falls within Barcode Index Number (BIN) BOLD:ACB4200, which also includes specimens from Italy and Germany, and is identical to a haplotype sequenced from Bulgaria. Two additional BINs on BOLD are associated with specimens identified as *A. scriptella*: BOLD:AAF3288, known from Germany and Austria and differing from OZ350385.1 by 3.5–3.8% *p*-distance, and BOLD:AGN9009, associated with sequence MF049938 from Hao *et al.* (2020), differing by about 3.26% *p*-distance and apparently representing material from China. Kızıldağ *et al.* (2019), in a study of DNA barcodes of the genus *Altenia* Sattler, 1960 in Türkiye, found that *A. scriptella* was the nearest neighbour of *Altenia anamuria* Kemal & Koçak, 2018, with a *p*-distance of 4.79–4.96%. On BOLD, DNA barcodes of *A. modesta* (Danilevsky, 1955) and *A. wagneriella* differ from OZ350385.1 by 4.4–4.8% *p*-distance.

*Altenia scriptella* represents the gelechiine tribe Litini Bruand, 1859 (= Teleiodini Piskunov, 1973). The genome will be useful for studying potential cryptic speciation and to clarify phylogenetic relationships of the genus *Altenia*, which has seven species worldwide. The Holarctic species *A. perspersella* (Wocke, 1862), the exemplar included in the phylogeny of Yapar *et al.* (2025), was recovered as sister to *Teleoides* Sattler, 1960 + ( *Pseudotelphusa* Janse, 1958 + *Teleiopsis* Sattler, 1960).

## Methods

### Sample acquisition and DNA barcoding

The specimen used for genome sequencing was an adult female *Altenia scriptella* (specimen ID NHMUK013698626, ToLID ilAltScri1; Figure 1), collected from Homefield Wood, Marlow, England, UK (latitude 51.57, longitude −0.83) on 2023-06-17. The specimen was collected and identified by David Lees (Natural History Museum).

The initial identification was verified by an additional DNA barcoding process according to the framework developed by Twyford *et al.* (2024). A small sample was dissected from the specimen and stored in ethanol, while the remaining parts were shipped on dry ice to the Wellcome Sanger Institute (WSI) (see the protocol). The tissue was lysed, the COI marker region was amplified by PCR, and amplicons were sequenced and compared to the BOLD database, confirming the species identification ( Crowley *et al.*, 2023). Following whole genome sequence generation, the relevant DNA barcode region was also used alongside the initial barcoding data for sample tracking at the WSI ( Twyford *et al.*, 2024). The standard operating procedures for Darwin Tree of Life barcoding are available on protocols.io.

### Nucleic acid extraction

Detailed protocols for nucleic acid extraction developed at the Wellcome Sanger Institute (WSI) Tree of Life Core Laboratory are available on protocols.io ( Howard *et al.*, 2025). The ilAltScri1 sample was weighed and triaged to determine the appropriate extraction protocol. For HMW DNA extraction, 5 mg of tissue from the whole organism was used. Tissue from the whole organism was homogenised by powermashing using a PowerMasher II tissue disruptor. High molecular weight (HMW) DNA was extracted in the WSI Scientific Operations core using the Automated MagAttract v2 protocol. Sheared DNA was purified by automated SPRI (solid-phase reversible immobilisation). The concentration of the sheared and purified DNA was assessed using a Nanodrop spectrophotometer and Qubit Fluorometer using the Qubit dsDNA High Sensitivity Assay kit. Fragment size distribution was evaluated by running the sample on the FemtoPulse system. For this sample, the final post-shearing DNA had a Qubit concentration of 13.23 ng/μL and a yield of 621.81 ng, with a fragment size of 15.0 kb. The Genomic Quality Number (GQN) was 7.5.

### PacBio HiFi library preparation and sequencing

Library preparation and sequencing were performed at the WSI Scientific Operations core. Libraries were prepared using the SMRTbell Prep Kit 3.0 (Pacific Biosciences, California, USA), following the manufacturer’s instructions. The kit includes reagents for end repair/A-tailing, adapter ligation, post-ligation SMRTbell bead clean-up, and nuclease treatment. Size selection and clean-up were performed using diluted AMPure PB beads (Pacific Biosciences). DNA concentration was quantified using a Qubit Fluorometer v4.0 (ThermoFisher Scientific) and the Qubit 1X dsDNA HS assay kit. Final library fragment size was assessed with the Agilent Femto Pulse Automated Pulsed Field CE Instrument (Agilent Technologies) using the gDNA 55 kb BAC analysis kit.

The sample was sequenced on a Revio instrument (Pacific Biosciences). The prepared library was normalised to 2 nM, and 15 μL was used for making complexes. Primers were annealed and polymerases bound to generate circularised complexes, following the manufacturer’s instructions. Complexes were purified using 1.2X SMRTbell beads, then diluted to the Revio loading concentration (200–300 pM) and spiked with a Revio sequencing internal control. The sample was sequenced on a Revio 25 M SMRT cell. The SMRT Link software (Pacific Biosciences), a web-based workflow manager, was used to configure and monitor the run and to carry out primary and secondary data analysis.

### Hi-C

***Sample preparation and crosslinking***

The Hi-C sample was prepared from 20–50 mg of frozen whole organism tissue from the ilAltScri1 sample using the Arima-HiC v2 kit (Arima Genomics). Following the manufacturer’s instructions, tissue was fixed and DNA crosslinked using TC buffer to a final formaldehyde concentration of 2%. The tissue was homogenised using the Diagnocine Power Masher-II. Crosslinked DNA was digested with a restriction enzyme master mix, biotinylated, and ligated. Clean-up was performed with SPRISelect beads before library preparation. DNA concentration was measured with the Qubit Fluorometer (Thermo Fisher Scientific) and Qubit HS Assay Kit. The biotinylation percentage was estimated using the Arima-HiC v2 QC beads.

***Hi-C library preparation and sequencing***

Biotinylated DNA constructs were fragmented using a Covaris E220 sonicator and size selected to 400–600 bp using SPRISelect beads. DNA was enriched with Arima-HiC v2 kit Enrichment beads. End repair, A-tailing, and adapter ligation were carried out with the NEBNext Ultra II DNA Library Prep Kit (New England Biolabs), following a modified protocol where library preparation occurs while DNA remains bound to the Enrichment beads. Library amplification was performed using KAPA HiFi HotStart mix and a custom Unique Dual Index (UDI) barcode set (Integrated DNA Technologies). Depending on sample concentration and biotinylation percentage determined at the crosslinking stage, libraries were amplified with 10–16 PCR cycles. Post-PCR clean-up was performed with SPRISelect beads. Libraries were quantified using the AccuClear Ultra High Sensitivity dsDNA Standards Assay Kit (Biotium) and a FLUOstar Omega plate reader (BMG Labtech).

Prior to sequencing, libraries were normalised to 10 ng/μL. Normalised libraries were quantified again to create equimolar and/or weighted 2.8 nM pools. Pool concentrations were checked using the Agilent 4200 TapeStation (Agilent) with High Sensitivity D500 reagents before sequencing. Sequencing was performed using paired-end 150 bp reads on the Illumina NovaSeq X.

### Genome assembly

Prior to assembly of the PacBio HiFi reads, a database of *k*-mer counts ( *k* = 31) was generated from the filtered reads using FastK. GenomeScope2 ( Ranallo-Benavidez *et al.*, 2020) was used to analyse the *k*-mer frequency distributions, providing estimates of genome size, heterozygosity, and repeat content.

The HiFi reads were assembled using Hifiasm in Hi-C phasing mode ( Cheng *et al.*, 2021, 2022), producing two haplotypes. Hi-C reads ( Rao *et al.*, 2014) were mapped to the primary contigs using bwa-mem2 ( Vasimuddin *et al.*, 2019). Contigs were further scaffolded with Hi-C data in YaHS ( Zhou *et al.*, 2023), using the --break option for handling potential misassemblies. The scaffolded assemblies were evaluated using Gfastats ( Formenti *et al.*, 2022), BUSCO ( Manni *et al.*, 2021) and MerquryFK ( Rhie *et al.*, 2020).

The mitochondrial genome was assembled using MitoHiFi ( Uliano-Silva *et al.*, 2023).

### Assembly curation

The assembly was decontaminated using the Assembly Screen for Cobionts and Contaminants ( ASCC) pipeline. TreeVal was used to generate the flat files and maps for use in curation. Manual curation was conducted primarily in PretextView and HiGlass ( Kerpedjiev *et al.*, 2018). Scaffolds were visually inspected and corrected as described by Howe *et al*. (2021). Manual corrections included six breaks and 90 joins. This reduced the scaffold count by 20.0% and increased the total assembly length by 4.3%. The curation process is described at https://gitlab.com/wtsi-grit/rapid-curation . PretextSnapshot was used to generate a Hi-C contact map of the final assembly.

### Assembly quality assessment

The MerquryFK tool ( Rhie *et al.*, 2020) was run in a Singularity container ( Kurtzer *et al.*, 2017) to evaluate *k*-mer completeness and assembly quality for both haplotypes using the *k*-mer database ( *k* = 31) computed prior to genome assembly. The analysis outputs included assembly QV scores and completeness statistics.

The genome was analysed using the BlobToolKit pipeline, a Nextflow implementation of the earlier Snakemake version ( Challis *et al.*, 2020). The pipeline aligns PacBio reads using minimap2 ( Li, 2018) and SAMtools ( Danecek *et al.*, 2021) to generate coverage tracks. It runs BUSCO ( Manni *et al.*, 2021) using lineages identified from the NCBI Taxonomy ( Schoch *et al.*, 2020). For the three domain-level lineages, BUSCO genes are aligned to the UniProt Reference Proteomes database ( Bateman *et al.*, 2023) using DIAMOND blastp ( Buchfink *et al.*, 2021). The genome is divided into chunks based on the density of BUSCO genes from the closest taxonomic lineage, and each chunk is aligned to the UniProt Reference Proteomes database with DIAMOND blastx. Sequences without hits are chunked using seqtk and aligned to the NT database with blastn ( Altschul *et al.*, 1990). The BlobToolKit suite consolidates all outputs into a blobdir for visualisation. The BlobToolKit pipeline was developed using nf-core tooling ( Ewels *et al.*, 2020) and MultiQC ( Ewels *et al.*, 2016), with containerisation through Docker ( Merkel, 2014) and Singularity ( Kurtzer *et al.*, 2017).

## Genome sequence report

### Sequence data

**Figure 2.**
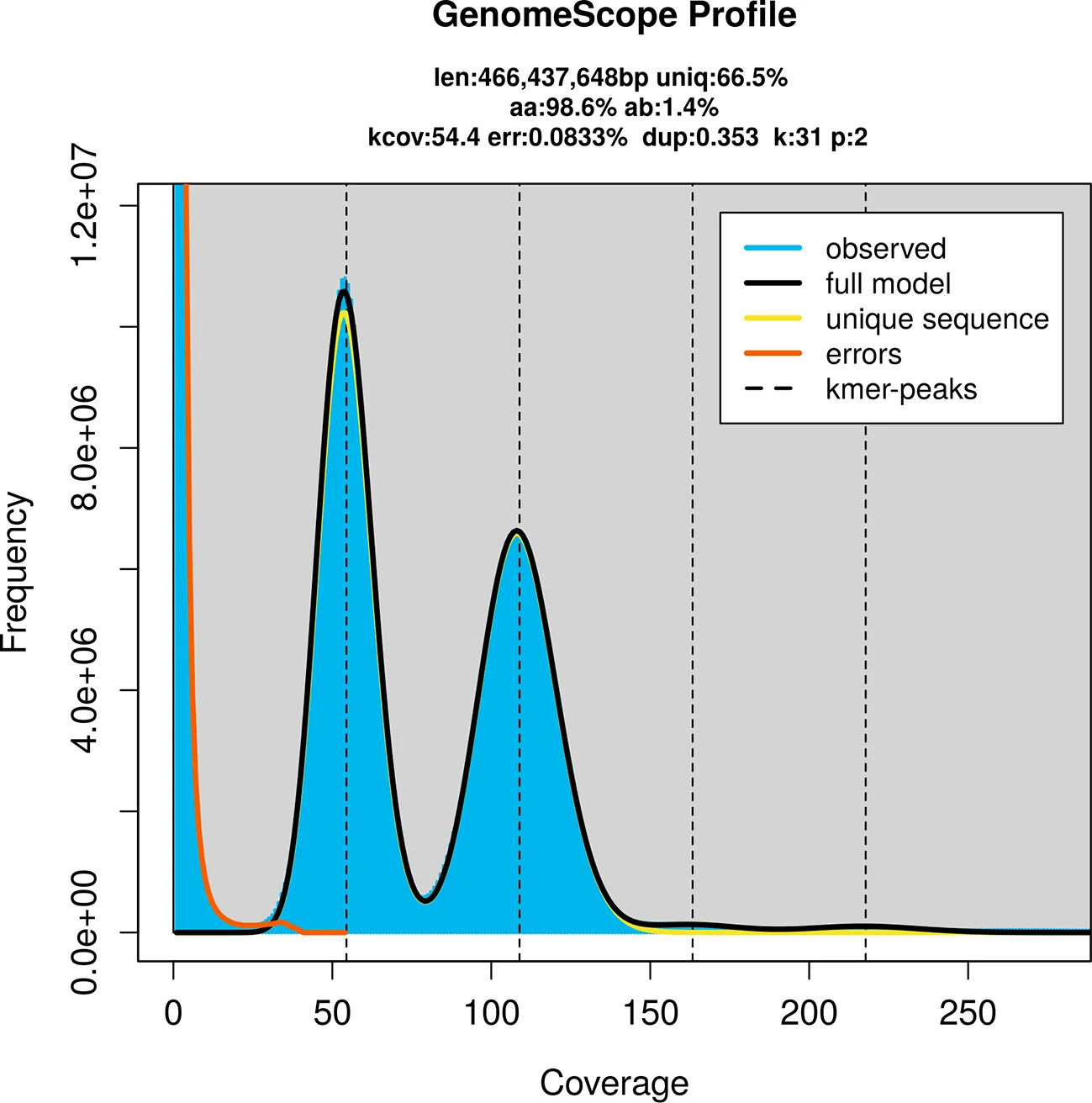
Frequency distribution of *k*-mers generated using GenomeScope2. The plot shows observed and modelled *k*-mer spectra, providing estimates of genome size, heterozygosity, and repeat content based on unassembled sequencing reads.

PacBio sequencing of the *Altenia scriptella* specimen generated 52.26 Gb (gigabases) from 4.64 million reads, which were used to assemble the genome. GenomeScope2.0 analysis estimated the haploid genome size at 466.71 Mb, with a heterozygosity of 1.40% and repeat content of 33.52% ( Figure 2). These estimates guided expectations for the assembly. Based on the estimated genome size, the sequencing data provided approximately 109× coverage. Hi-C sequencing produced 129.81 Gb from 429.82 million reads, which were used to scaffold the assembly. Table 1 summarises the specimen and sequencing details.

**Table 1.** Specimen and sequencing data for *Altenia scriptella* (BioProject PRJEB96572).

Platform	PacBio HiFi	Hi-C
**ToLID**	ilAltScri1	ilAltScri1
**Specimen ID**	NHMUK013698626	NHMUK013698626
**BioSample (source individual)**	SAMEA117491959	SAMEA117491959
**BioSample (tissue)**	SAMEA117492028	SAMEA117492028
**Tissue**	whole organism	whole organism
**Instrument**	Revio	Illumina NovaSeq X
**Run accessions**	ERR15499476	ERR15500435
**Read count total**	4.64 million	429.82 million
**Base count total**	52.26 Gb	129.81 Gb

### Assembly statistics

The genome was assembled into two haplotypes using Hi-C phasing. Haplotype 1 was curated to chromosome level, while haplotype 2 was assembled to scaffold level. The final assembly has a total length of 506.56 Mb in 99 scaffolds, with 79 gaps, and a scaffold N50 of 17.26 Mb ( Table 2).

**Table 2.** Genome assembly statistics for *Altenia scriptella.*

Genome assembly	Haplotype 1	Haplotype 2
**Assembly name**	ilAltScri1.hap1.1	ilAltScri1.hap2.1
**Assembly accession**	GCA_977013465.1	GCA_977013445.1
**Assembly level**	chromosome	scaffold
**Span (Mb)**	506.56	440.34
**Number of chromosomes**	33	scaffold-level
**Number of contigs**	178	89
**Contig N50**	16.14 Mb	16.08 Mb
**Number of scaffolds**	99	80
**Scaffold N50**	17.26 Mb	17.23 Mb
**Longest scaffold length (Mb)**	32.39	-
**Sex chromosomes**	W and Z	-
**Organelles**	Mitochondrion: 15.3 kb	-

**Figure 3.**
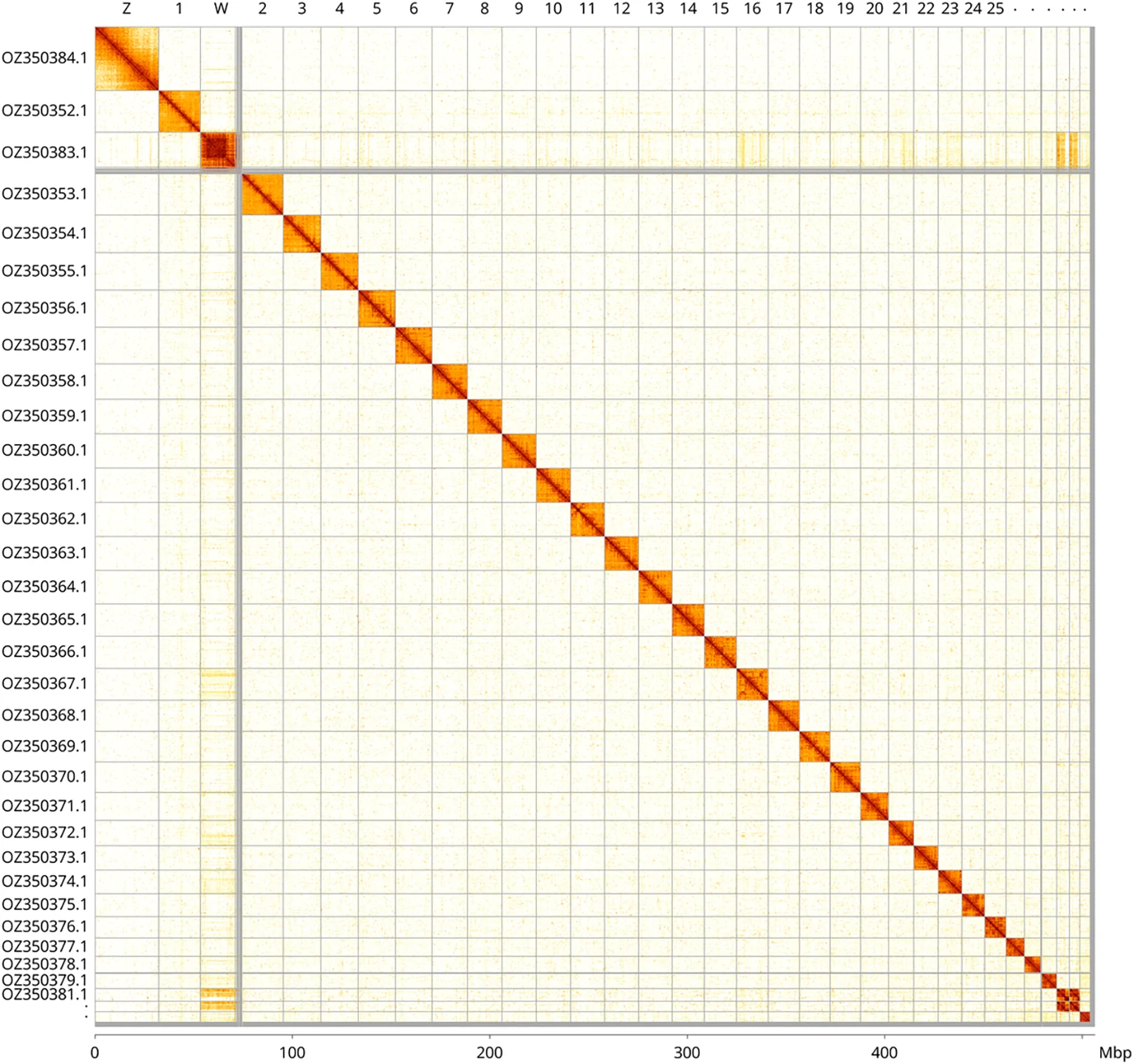
Hi-C contact map of the *Altenia scriptella* genome assembly. Assembled chromosomes are shown in order of size and labelled along the axes, with a megabase scale shown below. The plot was generated using PretextSnapshot.

Most of the haplotype 1 assembly sequence (99.51%) was assigned to 33 chromosomal-level scaffolds, representing 29 autosomes and the B1, B2, W, and Z sex chromosomes. These chromosome-level scaffolds, confirmed by Hi-C data, are named according to size ( Figure 3 and Table 3). The sex chromosomes were identified based on the copy number on the diploid HiC map. We identified two potential B chromosomes and both are in HAP1 file. We believe they are B chromosomes because: i) after BUSCO alignment to a close related species, all Altenia scriptella autosomes match one of the reference chromosomes, looking like that most of the content present in B chromosomes may be new to the genome; ii) all the few BUSCO hits present in the B chromosomes were duplicated and iii) similarity could be observed on the HiC map between the B chromosomes and some tiny regions of some of the autosomes, indicating they may have genes in common.

**Table 3.** Chromosomal pseudomolecules in the haplotype 1 genome assembly of *Altenia scriptella* ilAltScri1.

INSDC accession	Molecule	Length (Mb)	GC%
OZ350352.1	1	21.12	37.50
OZ350353.1	2	20.93	37
OZ350354.1	3	19.15	37.50
OZ350355.1	4	18.90	37.50
OZ350356.1	5	18.79	37
OZ350357.1	6	18.50	36.50
OZ350358.1	7	17.95	37
OZ350359.1	8	17.46	37
OZ350360.1	9	17.40	37
OZ350361.1	10	17.36	37
OZ350362.1	11	17.26	36.50
OZ350363.1	12	17.24	37
OZ350364.1	13	16.91	37.50
OZ350365.1	14	16.33	37
OZ350366.1	15	16.30	37
OZ350367.1	16	16.14	37.50
OZ350368.1	17	15.74	37
OZ350369.1	18	15.60	38
OZ350370.1	19	15.37	37.50
OZ350371.1	20	14.14	37.50
OZ350372.1	21	12.83	38
OZ350373.1	22	12.32	37
OZ350374.1	23	11.98	37.50
OZ350375.1	24	11.54	37.50
OZ350376.1	25	10.88	37
OZ350377.1	26	9.22	38
OZ350378.1	27	8.67	38.50
OZ350379.1	28	7.69	38
OZ350380.1	29	5.25	38.50
OZ350383.1	W	21.01	37.50
OZ350384.1	Z	32.39	37
OZ350381.1	B1	6.44	38
OZ350382.1	B2	5.26	38

The mitochondrial genome was also assembled (length 15.3 kb, OZ350385.1). This sequence is included as a contig in the multifasta file of the genome submission and as a standalone record.

### Assembly quality metrics

**Figure 4.**
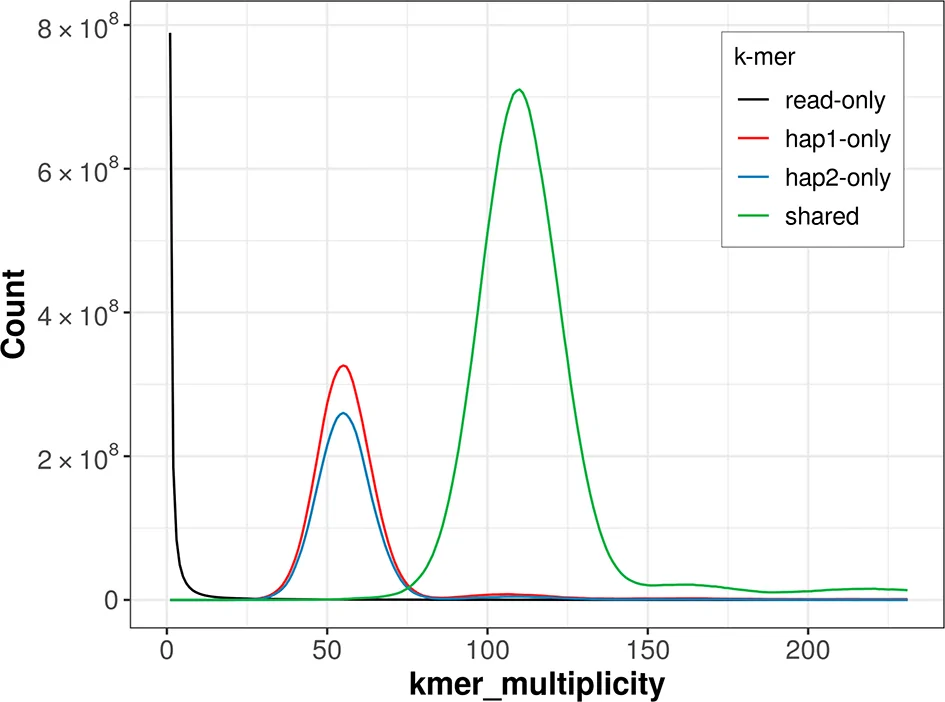
Evaluation of *k*-mer completeness using MerquryFK. This plot illustrates the recovery of *k*-mers from the original read data in the final assemblies. The horizontal axis represents *k*-mer multiplicity, and the vertical axis shows the number of *k*-mers. The black curve represents *k*-mers that appear in the reads but are not assembled. The green curve corresponds to *k*-mers shared by both haplotypes, and the red and blue curves show *k*-mers found only in one of the haplotypes.

For haplotype 1, the estimated QV is 68.0, and for haplotype 2, 69.0. When the two haplotypes are combined, the assembly achieves an estimated QV of 68.5. The *k*-mer completeness is 77.44% for haplotype 1, 71.32% for haplotype 2, and 99.70% for the combined haplotypes ( Figure 4).

**Figure 5.**
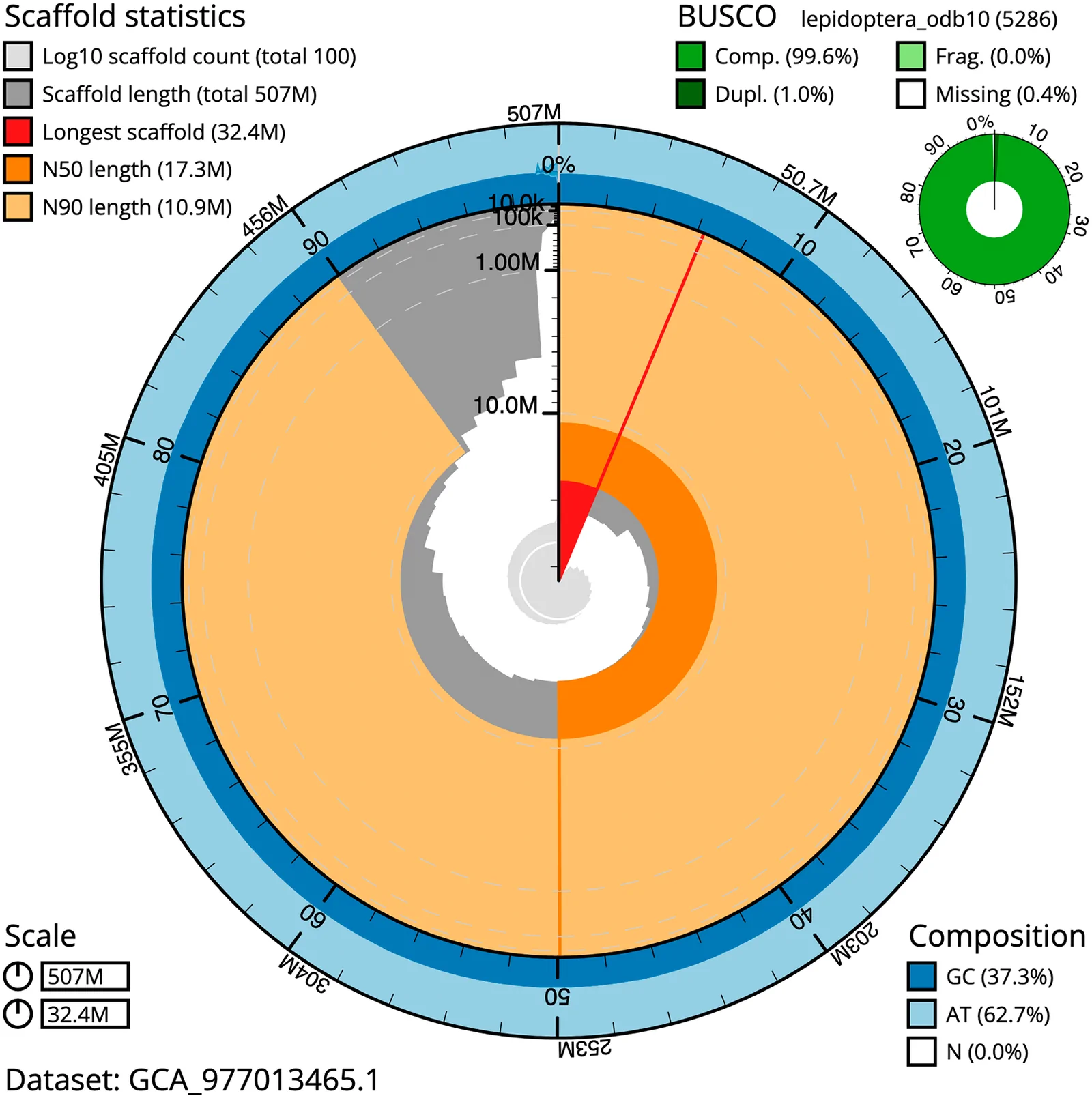
Assembly metrics for ilAltScri1.hap1.1. The BlobToolKit snail plot provides an overview of assembly metrics and BUSCO gene completeness. The circumference represents the length of the whole genome sequence, and the main plot is divided into 1 000 bins around the circumference. The outermost blue tracks display the distribution of GC, AT, and N percentages across the bins. Scaffolds are arranged clockwise from longest to shortest and are depicted in dark grey. The longest scaffold is indicated by the red arc, and the deeper orange and pale orange arcs represent the N50 and N90 lengths. A light grey spiral at the centre shows the cumulative scaffold count on a logarithmic scale. A summary of complete, fragmented, duplicated, and missing BUSCO genes in the set is presented at the top right. An interactive version of this figure can be accessed on the BlobToolKit viewer.

**Figure 6.**
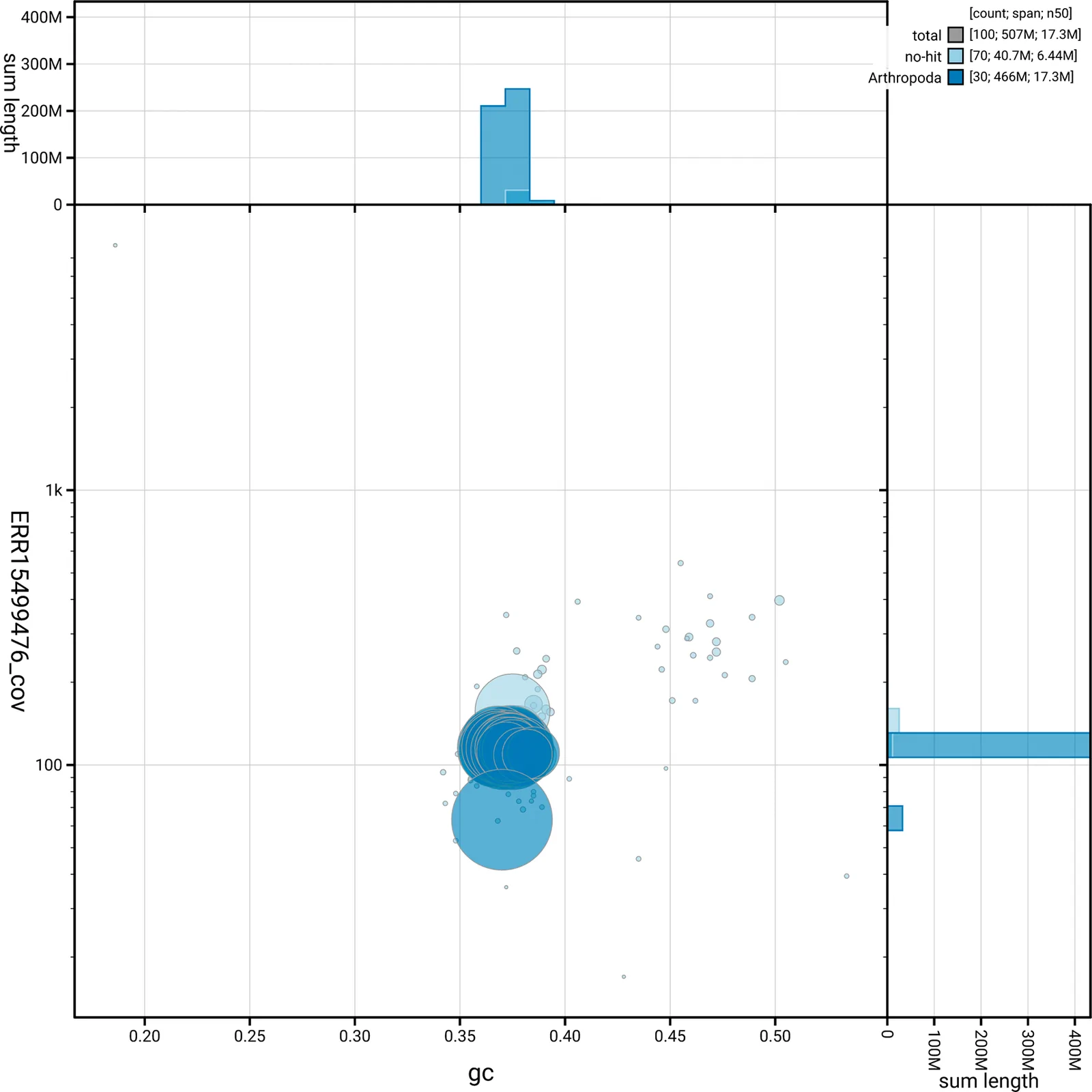
BlobToolKit blob plot for ilAltScri1.hap1.1. The plot shows base coverage (vertical axis) and GC content (horizontal axis). The circles represent scaffolds, with the size proportional to scaffold length and the colour representing phylum membership. The histograms along the axes display the total length of sequences distributed across different levels of coverage and GC content. An interactive version of this figure is available on the BlobToolKit viewer.

BUSCO analysis using the lepidoptera_odb10 reference set ( *n* = 5 286) identified 99.6% of the expected gene set (single = 98.6%, duplicated = 1.0%) in haplotype 1. For haplotype 2, BUSCO v.6.0.0 analysis identified 92.6% of the expected gene set (single = 91.8%, duplicated = 0.9%). The snail plot in Figure 5 summarises the scaffold length distribution and other assembly statistics for haplotype 1. The blob plot in Figure 6 shows the distribution of scaffolds by GC proportion and coverage for haplotype 1.

Table 4 lists the assembly metric benchmarks adapted from Rhie *et al.* (2021) and the Earth BioGenome Project Report on Assembly Standards January 2026. The EBP metric, calculated for the haplotype 1, is **7.C.Q68**, meeting the recommended reference standard.

**Table 4.** Earth Biogenome Project summary metrics for the *Altenia scriptella* assembly.

Measure	Value	Benchmark
EBP summary (haplotype 1)	7.C.Q68	6.C.Q40
Contig N50 length	16.14 Mb	≥ 1 Mb
Scaffold N50 length	17.26 Mb	= chromosome N50
Consensus quality (QV)	Haplotype 1: 68.0; haplotype 2: 69.0; combined: 68.5	≥ 40
*k*-mer completeness	Haplotype 1: 77.44%; Haplotype 2: 71.32%; combined: 99.70%	≥ 95%
BUSCO	C:99.6% [S:98.6%, D:1.0%], F:0.0%, M:0.4%, n:5 286	S > 90%; D < 5%
Percentage of assembly assigned to chromosomes	99.51%	≥ 90%

*Notes:* The EBP summary uses log10(Contig N50); chromosome-level (C) or log10(Scaffold N50); Q (Merqury QV). BUSCO: C = complete; S = single-copy; D = duplicated; F = fragmented; M = missing; n = orthologues.

**Table 5.** Software versions and sources used for *Altenia scriptella.*

Software	Version	Source
BLAST	2.14.0	ftp://ftp.ncbi.nlm.nih.gov/blast/executables/blast+/
BlobToolKit	4.4.6	https://github.com/blobtoolkit/blobtoolkit
BUSCO	6.0.0	https://gitlab.com/ezlab/busco
bwa-mem2	2.2.1	https://github.com/bwa-mem2/bwa-mem2
DIAMOND	2.1.8	https://github.com/bbuchfink/diamond
fasta_windows	0.2.4	https://github.com/tolkit/fasta_windows
FastK	1.1	https://github.com/thegenemyers/FASTK
GenomeScope2.0	2.0.1	https://github.com/tbenavi1/genomescope2.0
Gfastats	1.3.6	https://github.com/vgl-hub/gfastats
Hifiasm	0.25.0-r726	https://github.com/chhylp123/hifiasm
HiGlass	1.13.4	https://github.com/higlass/higlass
MerquryFK	1.1.2	https://github.com/thegenemyers/MERQURY.FK
Minimap2	2.28-r1209	https://github.com/lh3/minimap2
MitoHiFi	3	https://github.com/marcelauliano/MitoHiFi
MultiQC	1.14; 1.17 and 1.18	https://github.com/MultiQC/MultiQC
Nextflow	24.10.4	https://github.com/nextflow-io/nextflow
PretextSnapshot	0.0.5	https://github.com/sanger-tol/PretextSnapshot
PretextView	1.0.3	https://github.com/sanger-tol/PretextView
samtools	1.21	https://github.com/samtools/samtools
sanger-tol/ascc	0.1.0	https://github.com/sanger-tol/ascc
sanger-tol/blobtoolkit	v0.9.0	https://github.com/sanger-tol/blobtoolkit
sanger-tol/curationpretext	1.4.2	https://github.com/sanger-tol/curationpretext
Seqtk	1.3	https://github.com/lh3/seqtk
Singularity	3.9.0	https://github.com/sylabs/singularity
TreeVal	1.4.0	https://github.com/sanger-tol/treeval
YaHS	1.2.2	https://github.com/c-zhou/yahs

## Author information

Contributors are listed at the following links:

• Members of the Natural History Museum Genome Acquisition Lab
• Members of the Darwin Tree of Life Barcoding collective
• Members of the Wellcome Sanger Institute Tree of Life Management, Samples and Laboratory team
• Members of Wellcome Sanger Institute Scientific Operations – Sequencing Operations
• Members of the Wellcome Sanger Institute Tree of Life Core Informatics team
• Members of the Tree of Life Core Informatics collective
• Members of the Darwin Tree of Life Consortium

## Wellcome Sanger Institute – Legal and Governance

The materials that have contributed to this genome note have been supplied by a Darwin Tree of Life Partner. The submission of materials by a Darwin Tree of Life Partner is subject to the **‘Darwin Tree of Life Project Sampling Code of Practice’**, which can be found in full on the Darwin Tree of Life website. By agreeing with and signing up to the Sampling Code of Practice, the Darwin Tree of Life Partner agrees they will meet the legal and ethical requirements and standards set out within this document in respect of all samples acquired for, and supplied to, the Darwin Tree of Life Project. Further, the Wellcome Sanger Institute employs a process whereby due diligence is carried out proportionate to the nature of the materials themselves, and the circumstances under which they have been/are to be collected and provided for use. The purpose of this is to address and mitigate any potential legal and/or ethical implications of receipt and use of the materials as part of the research project, and to ensure that in doing so we align with best practice wherever possible. The overarching areas of consideration are:

• Ethical review of provenance and sourcing of the material
• Legality of collection, transfer and use (national and international)

Each transfer of samples is further undertaken according to a Research Collaboration Agreement or Material Transfer Agreement entered into by the Darwin Tree of Life Partner, Genome Research Limited (operating as the Wellcome Sanger Institute), and in some circumstances, other Darwin Tree of Life collaborators.

## Data Availability

European Nucleotide Archive: Altenia scriptella. Accession number PRJEB96572. The genome sequence is released openly for reuse. The *Altenia scriptella* genome sequencing initiative is part of the Darwin Tree of Life Project (PRJEB40665), the Sanger Institute Tree of Life Programme (PRJEB43745) and Project Psyche (PRJEB71705). All raw sequence data and the assembly have been deposited in INSDC databases. The genome will be annotated using available RNA-Seq data and presented through the Ensembl pipeline at the European Bioinformatics Institute. Raw data and assembly accession identifiers are reported in Tables 1 and 2. Production code used in genome assembly at the WSI Tree of Life is available at https://github.com/sanger-tol . Table 5 lists software versions used in this study.

## References

[ref1] Altschul SF Gish W Miller W : Basic Local Alignment Search Tool. *J. Mol. Biol.* 1990;215(3):403–410. 10.1016/S0022-2836(05)80360-2 2231712

[ref2] Bateman A Martin M-J Orchard S : UniProt: The Universal Protein Knowledgebase in 2023. *Nucleic Acids Res.* 2023;51(D1):D523–D531. 10.1093/nar/gkac1052 36408920 PMC9825514

[ref3] Bland KP Corley MFV Emmet AM : Gelechiidae. Emmet AM Langmaid JR , editors. *The Moths and Butterflies of Great Britain and Ireland.* Colchester: Harley Books;2002;4:277.

[ref4] Buchfink B Reuter K Drost H-G : Sensitive protein alignments at tree-of-life scale using DIAMOND. *Nat. Methods.* 2021;18(4):366–368. 10.1038/s41592-021-01101-x 33828273 PMC8026399

[ref5] Challis R Richards E Rajan J : BlobToolKit – interactive quality assessment of genome assemblies. *G3 Genes|Genomes|Genetics.* 2020;10(4):1361–1374. 10.1534/g3.119.400908 32071071 PMC7144090

[ref6] Cheng H Concepcion GT Feng X : Haplotype-resolved *de novo* assembly using phased assembly graphs with Hifiasm. *Nat. Methods.* 2021;18(2):170–175. 10.1038/s41592-020-01056-5 33526886 PMC7961889

[ref7] Cheng H Jarvis ED Fedrigo O : Haplotype-resolved assembly of diploid genomes without parental data. *Nat. Biotechnol.* 2022;40(9):1332–1335. 10.1038/s41587-022-01261-x 35332338 PMC9464699

[ref8] Crowley L Allen H Barnes I : A sampling strategy for genome sequencing the British terrestrial Arthropod fauna. *Wellcome Open Res.* 2023;8:123. 10.12688/wellcomeopenres.18925.1 37408610 PMC10318377

[ref9] Danecek P Bonfield JK Liddle J : Twelve years of SAMtools and BCFtools. *GigaScience.* 2021;10(2):giab008. 10.1093/gigascience/giab008 33590861 PMC7931819

[ref10] Ewels P Magnusson M Lundin S : MultiQC: Summarize analysis results for multiple tools and samples in a single report. *Bioinformatics.* 2016;32(19):3047–3048. 10.1093/bioinformatics/btw354 27312411 PMC5039924

[ref11] Ewels PA Peltzer A Fillinger S : The nf-core framework for community-curated bioinformatics pipelines. *Nat. Biotechnol.* 2020;38(3):276–278. 10.1038/s41587-020-0439-x 32055031

[ref12] Formenti G Abueg L Brajuka A : Gfastats: Conversion, evaluation and manipulation of genome sequences using assembly graphs. *Bioinformatics.* 2022;38(17):4214–4216. 10.1093/bioinformatics/btac460 35799367 PMC9438950

[ref13] GBIF Secretariat: *Altenia scriptella* (Hübner, 1796). 2025. Reference Source

[ref14] Gelechiidae Recording Scheme: 35.155 (766) *Altenia scriptella.* 2026. Reference Source

[ref15] Hao M Jin Q Guanliang M : Joint use of NGS and DNA barcoding facilitates community assembly inference and diversity assessment for species-rich taxon group: A case study on moth community of temperate forest ecosystem. *Evol. Ecol.* 2020;34:1063–1088. 10.1007/s10682-020-10072-y

[ref16] Howard C Denton A Jackson B : On the path to reference genomes for all biodiversity: Lessons learned and laboratory protocols created in the Sanger Tree of Life core laboratory over the first 2000 species. *bioRxiv.* 2025. 10.1101/2025.04.11.648334 PMC12548527 41129326

[ref17] Howe K Chow W Collins J : Significantly improving the quality of genome assemblies through curation. *GigaScience.* 2021;10(1):giaa153. 10.1093/gigascience/giaa153 33420778 PMC7794651

[ref18] Huemer P Karsholt O : *Gelechiidae i: (Gelechiinae: Teleiodini, Gelechiini).* Brill;2023; vol.3. 10.1163/9789004629028

[ref19] Kerpedjiev P Abdennur N Lekschas F : HiGlass: Web-based visual exploration and analysis of genome interaction maps. *Genome Biol.* 2018;19(1):125. 10.1186/s13059-018-1486-1 30143029 PMC6109259

[ref20] Kızıldağ S Kemel M Koçak AO : Molecular evaluations of some *Altenia* populations in Turkey (Lepidoptera, Gelechiidae). *Miscellaneous Papers Centre for Entomological Studies Ankara.* 2019;194:5–6.

[ref21] Kurtzer GM Sochat V Bauer MW : Singularity: Scientific containers for mobility of compute. *PLOS ONE.* 2017;12(5):e0177459. 10.1371/journal.pone.0177459 28494014 PMC5426675

[ref22] Li H : Minimap2: Pairwise alignment for nucleotide sequences. *Bioinformatics.* 2018;34(18):3094–3100. 10.1093/bioinformatics/bty191 29750242 PMC6137996

[ref23] Manni M Berkeley MR Seppey M : BUSCO update: Novel and streamlined workflows along with broader and deeper phylogenetic coverage for scoring of eukaryotic, prokaryotic, and viral genomes. *Mol. Biol. Evol.* 2021;38(10):4647–4654. 10.1093/molbev/msab199 34320186 PMC8476166

[ref24] Merkel D : Docker: Lightweight Linux containers for consistent development and deployment. *Linux J.* 2014;2014(239):2. 10.5555/2600239.2600241

[ref25] Norfolk Moths: *Altenia scriptella (Hübner,* 1796 *) Marbled Maple Moth.* 2025. Reference Source

[ref26] Ranallo-Benavidez TR Jaron KS Schatz MC : GenomeScope 2.0 and Smudgeplot for reference-free profiling of polyploid genomes. *Nat. Commun.* 2020;11(1):1432. 10.1038/s41467-020-14998-3 32188846 PMC7080791

[ref27] Rao SSP Huntley MH Durand NC : A 3D map of the human genome at kilobase resolution reveals principles of chromatin looping. *Cell.* 2014;159(7):1665–1680. 10.1016/j.cell.2014.11.021 25497547 PMC5635824

[ref28] Rhie A McCarthy SA Fedrigo O : Towards complete and error-free genome assemblies of all vertebrate species. *Nature.* 2021;592(7856):737–746. 10.1038/s41586-021-03451-0 33911273 PMC8081667

[ref29] Rhie A Walenz BP Koren S : Merqury: Reference-free quality, completeness, and phasing assessment for genome assemblies. *Genome Biol.* 2020;21(1):245. 10.1186/s13059-020-02134-9 32928274 PMC7488777

[ref30] Schoch CL Ciufo S Domrachev M : NCBI taxonomy: A comprehensive update on curation, resources and tools. *Database.* 2020;2020:baaa062. 10.1093/database/baaa062 32761142 PMC7408187

[ref31] Sterling P Parsons M Lewington R : *Field Guide to the Micro-Moths of Great Britain and Ireland.* London: Bloomsbury Publishing;2023.

[ref32] Twyford AD Beasley J Barnes I : A DNA barcoding framework for taxonomic verification in the Darwin Tree of Life Project. *Wellcome Open Res.* 2024;9:339. 10.12688/wellcomeopenres.21143.1 39386966 PMC11462125

[ref33] Uliano-Silva M Ferreira JGRN Krasheninnikova K : MitoHiFi: A Python pipeline for mitochondrial genome assembly from PacBio high fidelity reads. *BMC Bioinformatics.* 2023;24(1):288. 10.1186/s12859-023-05385-y 37464285 PMC10354987

[ref34] Vasimuddin M Misra S Li H : Efficient architecture-aware acceleration of BWA-MEM for multicore systems. *2019 IEEE International Parallel and Distributed Processing Symposium (IPDPS).* IEEE;2019;314–324. 10.1109/IPDPS.2019.00041

[ref35] Yapar E Chiocchio A Heikkilä M : Integrating Sanger and next-generation sequencing data sheds light on phylogenetic relationships among gelechioid moths (Lepidoptera: Gelechioidea). *Syst. Entomol.* 2025;51:e70009. 10.1111/syen.70009

[ref36] Zhou C McCarthy SA Durbin R : YaHS: Yet another Hi-C scaffolding tool. *Bioinformatics.* 2023;39(1):btac808. 10.1093/bioinformatics/btac808 36525368 PMC9848053

