# Supplementary material for: The genome sequence of the Marbled Maple moth, Altenia scriptella (Hübner, 1796) (Lepidoptera: Gelechiidae)
Source: Wellcome Open Res. 2026 Jul 11;11:432. [Version 1] doi: 10.12688/wellcomeopenres.26552.1 (PMC13560575; doi:10.12688/wellcomeopenres.26552.1)
Supplement: Peer review history

# Reviewer report: Reviewer response for version 1

**DOI:** 10.21956/wellcomeopenres.29243.r161832

**Published:** 2026-08-13

**Authors:** Sean T S Law

**Affiliations:** 1 Independent Researcher, formerly at The Chinese University of Hong Kong, Hong Kong, China

This data descriptor presents a high-quality genome assembly of a female Marbled Maple moth. The assembly is of high completeness (a BUSCO score of 99.6%) and contiguity (Scaffold N50 of 17Mb).

 In addition to 29 autosomes, Z and W sex chromosomes, two B chromosomes were reported in the Hap1 assembly.

 However, the rationale (namely (i) 1-to-1 relationship of BUSCO alignments are found between autosomes of Marbled Maple moth and its closely related species, and (ii) BUSCO hits on potential B chromosomes are duplicated) may not rule out the possibility that the one or both two potential B chromosome(s) is/are actually part of the W chromosome but failed to join during scaffolding.

 Notably, there are substantial interactions between the potential B chromosomes and the W chromosome in the contact map (though this could stem from noise in highly repetitive regions). As the Z and W sex chromosomes were identified based on the copy number on the diploid HiC map, presenting the data of the coverage / copy number of HiC data on all scaffolds may help clarify the distinction between the W chromosome and potential B chromosomes.

Are sufficient details of methods and materials provided to allow replication by others?

Yes

Is the rationale for creating the dataset(s) clearly described?

Yes

Are the datasets clearly presented in a useable and accessible format?

Yes

Are the protocols appropriate and is the work technically sound?

Yes

Reviewer Expertise:

Evolutionary biology, comparative genomics

I confirm that I have read this submission and believe that I have an appropriate level of expertise to confirm that it is of an acceptable scientific standard.

# Reviewer report: Reviewer response for version 1

**DOI:** 10.21956/wellcomeopenres.29243.r161824

**Published:** 2026-07-28

**Authors:** James Mallet

**Affiliations:** 1 Harvard University, Cambridge, Massachusetts, USA

A new highly contiguous genome assembly has been obtained for this species of Gelechiidae moth, *Altenia scriptella*. There are 33 chromosomes, which equates to the canonical 31 chromosomes of most Lepidoptera, except that there are two B chromosomes (assumed to be relatively non-functional). The statistics for BUSCO genes (generally considered important and shared across the tree of life) are particularly impressive:  99.6%.

Are sufficient details of methods and materials provided to allow replication by others?

Yes

Is the rationale for creating the dataset(s) clearly described?

Yes

Are the datasets clearly presented in a useable and accessible format?

Yes

Are the protocols appropriate and is the work technically sound?

Yes

Reviewer Expertise:

evolutionary genomics, particularly Lepidoptera

I confirm that I have read this submission and believe that I have an appropriate level of expertise to confirm that it is of an acceptable scientific standard.
